# Association of Hepatitis B Virus Pre-S Deletions with the Development of Hepatocellular Carcinoma in Qidong, China

**DOI:** 10.1371/journal.pone.0098257

**Published:** 2014-05-21

**Authors:** Li-Shuai Qu, Jin-Xia Liu, Tao-Tao Liu, Xi-Zhong Shen, Tao-Yang Chen, Zheng-Pin Ni, Cui-Hua Lu

**Affiliations:** 1 Department of Gastroenterology, Affiliated Hospital of Nantong University, Jiangsu Province, China; 2 Department of Gastroenterology, Zhongshan Hospital, Fudan University, Shanghai, China; 3 Qidong Liver Cancer Institute, Qidong, Jiangsu Province, China; CRCL-INSERM, France

## Abstract

**Background/Aim:**

To investigate the roles of mutations in pre-S and S regions of hepatitis B virus (HBV) on the progression of hepatocellular carcinoma (HCC) in Qidong, China.

**Methods:**

We conducted an age matched case-control study within a cohort of 2387 male HBV carriers who were recruited from August, 1996. The HBV DNA sequence in pre-S/S regions was successfully determined in 96 HCC cases and 97 control subjects. In addition, a consecutive series of samples from 11 HCC cases were employed to evaluate the pre-S deletion patterns before and after the occurrence of HCC.

**Results:**

After adjustment for age, history of cigarette smoking and alcohol consumption, HBeAg positivity, pre-S deletions, pre-S2 start codon mutations, and T53C mutation were significantly associated with HCC, showing adjusted odds ratios (ORs) from 1.914 to 3.199. HCC patients also had a lower frequency of T31C mutation in pre-S2 gene, compared with control subjects (0.524; 95% CI 0.280-0.982). HBV pre-S deletions were clustered mainly in the 5′ end of pre-S2 region. Multivariate analysis showed that pre-S deletions and pre-S2 start codon mutations were independent risk factors for HCC. The OR (95% CI) were 2.434 (1.063–5.573) and 3.065 (1.099–8.547), respectively. The longitudinal observation indicated that the pre-S deletion mutations were not acquired at the beginning of HBV infection, but that the mutations occurred during the long course of liver disease.

**Conclusion:**

Pre-S deletions and pre-S2 start codon mutations were independently associated with the development of HCC. The results also provided direct evidence that pre-S deletion mutations were not acquired from the beginning of infection but arose de novo during the progression of liver disease.

## Introduction

Hepatitis B virus (HBV) infection is a global health problem. Although an effective vaccine has been used for two decades, more than 350 million people in the world are chronic carriers of this virus [1], [2]. It is generally accepted that HBV played a major causative role in the development of hepatocellular carcinoma (HCC) in humans [3], [4]. Up to 80% of HCC is caused by HBV infection. However, the oncogenic mechanisms of HBV remain elusive. With the huge demand for HCC surveillance in China, where the prevalence of HBV infection is high, identification of risk factors for HCC and stratification of patient risk are very important to guide future surveillance strategy.

HBV has a partially double-stranded DNA genome of about 3.2 kb with four overlapping open reading frames (ORFs): the X, precore/core, pre-S/S, and Pol regions [5]. HBV replicates through RNA-intermediated reverse transcription. Because reverse transcriptase lacks proofreading activity, errors in HBV replication occur at a much higher rate than in other DNA viruses. Recently, viral mutations associated with the development of HCC have become a major focus for research. The most convincing association between viral mutation and the development of HCC is T1762/A1764 double mutations in the basal core promoter (BCP). BCP double mutations were confirmed to be associated with HCC in two large prospective cohort studies [6], [7]. Additionally, T1766, A1768, V1753 in BCP and T1653 mutations in box-α of Enhancer II have been found to be associated with the development of HCC in several reports [8]–[11]. Previous studies demonstrated that a number of pre-S1/S2 rearrangements, including deletions and start codon mutations, accumulated in patients at the later stage of chronic HBV infection and during fulminant hepatitis [12], [13]. Recent cross-sectional studies have also demonstrated that patients with progressive liver diseases had a higher frequency of pre-S deletions [14], [15]. However, the relationship between the pattern of pre-S deletions and the development of HCC has not been thoroughly investigated. Furthermore, current knowledge concerning the role of other HBV mutations in pre-S or S regions in HCC is limited. In addition, distinct clinical and virological characteristics of the HBV infection have been reported in different geographical parts of the world and are increasingly associated with genetic diversity of the infecting virus [16]. The data are largely lacking in mainland of China, where chronic HBV infection is highly endemic and accounts for half of the chronic hepatitis B infections in the world.

The township of Qidong is one of the highest endemic regions for chronic HBV infection and HCC in China. This case-control study was conducted within a large cohort of male HBV carriers in Qidong. In this study, we sought to investigate the prevalence of pre-S/S mutations on the development of HCC.

## Methods

### Patients and samples

The analysis used data and stored samples from a prospective cohort in Qidong, Jiangsu Province, China. The enrollment of the study cohort has been described previously [17]–[19]. Briefly, a total of 2387 males living in 17 townships of Qidong who were seropositive for hepatitis B surface antigen (HBsAg) and free of HCC at recruitment were followed up from 1996 to October 2006. Study participants were scheduled to undergo ultrasonography measurements, serum alpha-fetoprotein (AFP) level and conventional liver function tests every 6–12 months. The diagnosis of HCC was based on the following criteria: a histopathological examination; 1 imaging technique and a serum AFP level ≥400 ng/mL; or a positive lesion detected by at least 2 different imaging techniques (US, CT, MRI, and hepatic angiography). Several cases qualified based on more than 1 criterion. For this case-control study, we recruited 100 HCC patients and 100 chronic hepatitis (CH) patients as controls from the cohort of HBsAg carriers who were alive and had not been diagnosed with HCC throughout the follow-up period. All these 200 participants were positive for HBsAg and HBV DNA. The controls were individually matched to the cases by age (within 2 years). Subjects were excluded if they had poor sequence data (2 cases and 1 control) or a history of antiviral therapy (2 cases and 2 controls). Consequently, a total of 96 cases and 97 controls were included in the analysis. At recruitment, each study participant provided informed written consent and a structured questionnaire on sociodemographic characteristics, habits of alcohol and tobacco consumption. Serum samples collected at interview were stored at −70°C before analysis. This study was approved by the research ethics committee at Zhongshan Hospital, Fudan University, Shanghai, China.

### Serology

Serum HBsAg, HBeAg, and hepatitis C virus (HCV) antibody were tested by commercially available enzyme immunoassay kits (Shanghai Kehua Bio-engineering Co. Ltd., China). Serum alanine aminotransferase (ALT) level was determined by ultraviolet-lactate dehydrogenase (UVLDH) method (Shanghai Kehua Bio-engineering Co. Ltd). The serum HBV DNA levels were determined using the Fluorescein quantitative polymerase chain reaction (FQ-PCR) detection system (Taqmen; Roche US), according to the manufacturer's instructions. The lower limit of detection was 100 IU/mL.

### Nested polymerase chain reaction and direct sequencing of the pre-S/S regions

HBV DNA was extracted from 200 µL serum samples using the commercial Kit (Shanghai Shenyou Biotech Company, China). HBV genes of the pre-S/S regions were amplified by nested PCR. First-round PCR primers were 5′-AAAATTAATTA TCCTGCTAGG-3′ (nt 2627–2648) and 5′-CCCAAAAGACCCAAATTC-3′ (nt 1013–995). PCR reaction was carried out in 50 µL containing 5 µL 10 × buffer, 4 µL 2.5 mmol/L deoxynucleoside triphosphates (dNTP), 2 µL 10 µmol/L sense and antisense primers, 1.5 U PlatinumTaq DNA polymerase (Invitrogen, shanghai, China). First- round PCR was performed as follows: 95°C for 2 min; 95°C for 30 sec, 56°C for 30 sec, and 68°C for 3 min for 35 cycles; and finally, 68°C for 10 min. 2 µL of the first-round PCR product was reamplified by the same PCR condition as the first-round reaction. Second-round PCR primers were 5′-TTTACAACTCTGT GGAAGGC-3′ (nt 2747–2767) and 5′-CCAATACATATCCCATGAACT-3′ (nt 893–873). All necessary precautions to prevent cross-contamination were taken, and negative controls (no DNA added) were included in each assay. One negative control was included every 7 clinical samples from DNA extraction and PCR amplification. The amplicons were isolated by 2% agarose gel electrophoresis and purified by ethanol precipitation. Both strands of PCR products were directly sequenced in the forward and reverse directions using an ABI 3700 sequencer and commercial kit (Applied Biosystems, Foster City, CA).

### HBV genotyping

HBV genotyping was determined by phylogenetic analysis using the sequences of HBV pre-S/S regions. The compared standard genome sequences were downloaded from GenBank. Nucleotide sequences of HBV were multiple-aligned by the Clustal X program. The genetic distances were estimated by Kimura's two-parameter method, and the phylogenetic trees were constructed by the neighbor-joining method. To confirm the reliability of the phylogenetic tree analysis, bootstrap re-sampling and reconstruction with 1000 replicates were used. These analyses were carried out using software MEGA version 3.1.

### Statistical analysis

Data are presented as means ± SD, proportions, or median (range). To compare the values between the two groups, χ^2^ or Fisher exact tests were performed for categorical variables and the Student's t-test and nonparametric test were used for continuous variables where they were appropriate, respectively. Binary unconditional logistic regression models were used to estimate the odds ratios (ORs) of HCC associated with HBV-related factors and corresponding 95% confidence intervals (CIs). Potential confounders including age, history of cigarette smoking and alcohol consumption were adjusted. Multivariate analyses with stepwise logistic regression were used to determine the independent factors associated with HCC. The original data source were transferred to Microsoft Excel 2003 for analysis and attached as Supplementary Data (Table S1). All statistical tests were two sided. *P*<0.05 was considered statistically significant. All statistical analyses were performed using SPSS 11.5 for Windows (SPSS Inc., Chicago, IL).

## Results

### Clinical features and virologic characteristics of HCC patients and controls

The demographic data of the 96 HCC patients and 97 control subjects were listed in Table 1. There were no statistically significant differences in age, the histories of cigarette smoking and alcohol consumption between HCC patients and controls. The patients with HCC had significantly higher HBeAg positivity (P = 0.041). However, serum HBV DNA level was not different between patients with HCC and controls (P>0.05). After adjustment for age, history of cigarette smoking and alcohol consumption, the OR for ALT elevation (>45 IU/L) was 1.296 (95% CI, 0.657–2.556); seropositivity for HBeAg, 1.914 (95% CI, 1.026–3.570); high HBV DNA levels (>2000 IU/ml), 1.065 (95%, 0.602–1.882). Genotype C dominated the HBV types in Qidong, accounting for 92.7% of HCC patients and 94.8% control subjects. All of the genotype C strains isolated from Qidong were of the subgenotype C2, and all of the genotype B strains were of the subgenotype B2. There was no difference in genotype distribution between the HCC and control groups (P>0.05). The number of substitutions in pre-S/S regions was calculated after comparing with each corresponding prototype sequence from GenBank (Version GI289976889 for genotype C and GI289976881 for genotype B, both from an HBV carrier in Qidong, China). Table 1 also showed the number of nucleotide substitution in pre-S/S regions of HBV. The HCC group had significantly more nucleotide substitutions in the pre-S2 region (P = 0.007). The pre-S1 and S genes only showed slightly increased nucleotide substitutions in the HCC compared with the non-HCC group (P = 0.123 and P = 0.173, respectively).

**Table 1:** Characteristics of HCC patients and control subjects.

	HCC patients	Controls	Adjusted odds ratio*	
Variable	n = 96 (%)	n = 97 (%)	(95% CI)	*P*-value
Age (yr)	45.0 ± 8.3	44.2 ± 9.5	-	0.537
Cigarette smoking	50 (52.1)	46 (47.4)	-	0.517
Alcohol consumption	59 (61.5)	56 (57.7)	-	0.598
ALT>45 IU/L	24 (25.0)	20 (20.6)	1.296 (0.657–2.556)	0.455
HBeAg positivity	37 (38.5)	24 (24.7)	1.914 (1.026–3.570)	0.041
HBV DNA levels				
>2000 IU/ml	45 (46.9)	44 (45.4)	1.065 (0.602–1.882)	0.829
Genotype C	89 (92.7)	92 (94.8)	0.739 (0.219–2.497)	0.627
Number of mutations				
Pre-S1 (nts 2848–3204)	7.73 ± 3.06	7.07 ± 2.72	1.082 (0.979–1.195)	0.123
Pre-S2 (nts 3205–154)	11.75 ± 5.15	9.75 ± 4.74	1.088 (1.024–1.155)	0.007
S (nts 155–835)	5.57 ± 1.98	5.21 ± 2.11	1.103 (0.958–1.271)	0.173

ALT, alanine aminotransferase; HBeAg, hepatitis B e antigen;

*Adjusted for age, history of cigarette smoking, history of alcohol consumption.

### Identification of HCC-related mutations in pre-S/S regions

Table 2 lists all the mutations with frequencies of greater than 10% were observed in the pre-S/S genome of HBV. These included well-studied mutations (e.g., the pre-S2 start codon mutation and pre-S deletion) and less well-defined mutations (e.g., C3026A or T in pre-S1, T31C and T53C in pre-S2, A162G, T531C or G, A706C, and T766A in S). Among these nine hot spot mutations, pre-S deletions, pre-S start codon mutations, T31C, and T53C mutations were significantly associated with HCC, showing adjusted ORs from 0.524 to 3.199 (Table 2). These four mutations were all located in the pre-S gene. Although the S gene constitutes 21.2% of the entire HBV genome, there was no mutation in the S gene that showed a significantly different frequency in the HCC group. These data suggested that HCC-related mutations were not likely to distribute evenly throughout the HBV genome.

**Table 2:** Association between HCC and the presence of specific mutations in Pre-S/S regions.

		Total	HCC	Controls	Adjusted odds ratio*	
Mutation	Amino acid	n = 193 (%)	n = 96 (%)	n = 97 (%)	(95% CI)	*P*-value
Pre-S deletion	-	39 (20.2)	28 (29.2)	11 (11.3)	3.199 (1.483–6.900)	0.003
Pre-S1 gene						
C3026A/T	A 60 V/E	32 (16.6)	19 (19.8)	13 (13.4)	1.657 (0.760–3.610)	0.204
Pre-S2 gene						
Pre-S2 start codon	M 1 V/T/I	24 (12.4)	17 (17.7)	7 (7.2)	2.959 (1.132–7.736)	0.027
T31C	D 15 (no change)	60 (31.1)	23 (24.0)	37 (38.1)	0.524 (0.280–0.982)	0.044
T53C	F 22 L	59 (30.6)	36 (37.5)	23 (23.7)	2.043 (1.083–3.857)	0.027
Surface gene						
A162G	N 3 S	58 (30.1)	31 (32.3)	27 (27.8)	1.226 (0.656–2.290)	0.523
T531C/G	I 126 T/S	30 (15.5)	18 (18.8)	12 (12.4)	1.665 (0.748–3.704)	0.211
A706C	V 184 A	21 (10.9)	12 (12.5)	9 (9.3)	1.482 (0.583–3.769)	0.409
T766A	S 204 R	27 (14.0)	13 (13.5)	14 (14.4)	0.982 (0.428–2.255)	0.966

*Adjusted for age, history of cigarette smoking, history of alcohol consumption.

### Deletion patterns in the pre-S region

The location and size of pre-S deletions were present in table 3. Of the 39 patients with pre-S deletions, the types of pre-S deletion mutations could be categorized into 5 major types according to the deletion site: type I [5 HCC patients and 2 control subjects; pre-S1 deletion (N-half predominant; range, aa 1–57)]; type II [3 HCC patients and 1 control subject; pre-S1 deletion (C-half predominant; range, aa 58–119)]; type III (13 HCC patients and 6 control subjects; pre-S2 deletion only; type IV (2 HCC patients and 1 control subject; border deletion between pre-S1 and pre-S2 regions; type V (1 HCC patient; deleted at 2 separated sites, one in the pre-S1 region, the other in the pre-S2 region); and combined deletion type (4 HCC patients and 1 control subject; unclassified). Overall, the length of pre-S deletions varied from 6 to 84 bps. HBV pre-S deletions were clustered mainly in the 5′ end of pre-S2 region [19 (48.7%) of 39]. Pre-S deletions were more often found between amino acids 120 and 142 of the pre-S2 domain. Loss of pre-S2 start codon was identified in 4 HCC patients (14.2%) and 1 control subject (9.1%). Among the 39 HBV isolates with pre-S deletions identified in both HCC and control groups, 36 (92.3%) belonged to HBV genotype C. Most of the deletion regions encompassed T cell and B cell epitopes and important functional sites.

**Table 3:** Characteristics of pe-S deletions in 28 HCC patients and 11 control subjects.

					Size of	Deletion
Case	Age(y)	Genotype	Region (aa)	Region (nts)	deletion (bp)	Type*
**HCC patients**						
#3	45	C	131–140	22–54	33	III
#7	52	C	101–115	3148–3195	48	II
#8	58	C	2–6	2849–2866	18	I
#11	39	C	127–140	11–55	45	III
#13	41	C	106–128	3161–14	69	IV
#19	45	C	80–87	3085–3108	24	II
#24	50	C	20–26	2905–2925	21	
			96–122	3132–3215	84	I+IV
#26	47	C	133–140	28–54	27	III
#30	56	C	128–140	14–55	42	III
#33	58	C	102–121	3150–3212	63	
			139–140	47–55	9	III+IV
#41	57	C	132–141	25–57	33	III
#45	36	C	7–11	2864–2882	19	I
#52	50	C	2–5	2849–2863	15	I
#55	31	C	143–150	57–83	27	III
#62	59	C	21–24	2906–2920	15	I
#64	62	C	132–139	24–50	30	III
#69	60	B	21–25	2906–2923	18	
			81–107	3088–3168	81	I+II
#74	57	C	21–24	2906–2920	15	
			132–140	26–55	30	I+III
#76	49	C	139–140	47–55	9	III
#79	48	C	127–140	11–55	45	III
#80	44	C	132–140	26–55	30	III
#84	52	C	25–27	2920–2929	10	
			31–36	2936–2955	20	I
#85	39	C	116–125	3195–8	29	IV
#89	47	C	138–140	43–54	12	III
#91	42	B	63–88	3032–3112	81	
			131–137	22–45	24	V
#92	51	C	144–158	61–108	48	III
#95	44	C	62–68	3029–3052	24	II
#96	45	C	132–140	26–55	30	III
**Control subjects**						
#5	52	C	133–140	28–54	27	III
#14	44	C	116–141	3193–57	80	IV
#17	61	B	132–141	25–57	33	III
#28	48	C	46–49	2981–2995	15	I
#35	54	C	125–136	5–40	36	III
#39	46	C	2–5	2849–2863	15	
			102–116	3150–3196	47	I+II
#56	60	C	125–140	4–54	51	III
#75	38	C	24–32	2917–2944	28	I
#84	44	C	126–139	7–51	45	III
#89	49	C	101–116	3146–3196	51	II
#94	51	C	140	49–54	6	III

aa, amino acid; bp, base pair; nt, nucleotide.

*Deletion mutations are divided into the following 5 types: type I, pre-S1 deletion (N-half predominant; range, aa 1–57); type II, pre-S1 deletion (C-half predominant; range, aa 58–119); type III, pre-S2 deletion only; type IV, border between pre-S1 and pre-S2 region; type V, deleted at 2 separated sites, one in the pre-S1 region, the other in the pre-S2 region.

### Multivariate analysis on the risk factors for HCC

Unconditional logistic regression analyses showed that HBeAg positivity, and four sequence mutations (listed in Table 2) were significantly associated the subsequent risk of HCC. On further calculation using stepwise logistic regression analysis, the followings were found to be independent risk factors of HCC: pre-S deletions and pre-S2 start codon mutations (Table 4).

**Table 4:** Multivariate analysis of independent factors for the risk of HCC.

Factors	Odds ratio (95% CI)	*P*-value
Pre-S deletion	2.434 (1.063–5.573)	0.035
Pre-S2 start codon mutation	3.065 (1.099–8.547)	0.032

### Longitudinal observation of HBV pre-S deletion in HCC patients

Most previous studies on the relationship between HBV mutation and HCC were conducted with a single blood sample. In this study, we also examined the HBV mutations in serum samples spanning the years before and after HCC diagnosis. Among 28 HCC patients with pre-S deletion mutations, 11 HCC patients with sequential serum samples were selected for the longitudinal investigation of specific mutations (pre-S1 and pre-S2 deletion mutations). Table 5 demonstrated the evolution of pre-S deletion mutations during the progression of HCC. The lack of some information was due to the negative PCR product. There were 11 HCC patients whose HBV sequence could be determined from the serum samples collected at recruitment. Of these 11 HCC patients, 3 (27.3%) harbored HBV with only pre-S1 deletions, 6 (54.5%) harbored HBV with only pre-S2 deletions, and 2 (18.2%) harbored HBV with both pre-S1 and pre-S2 deletions. Among these 11 patients, 6 patients showed a gradual occurrence of pre-S1 and pre-S2 deletion mutations during follow-up. Reverse mutation was not observed in any patient. These results, together with those from our case-control study, indicated that the pre-S deletion mutations were not acquired at the beginning of HBV infection, but that the mutations occurred during the long course of liver disease.

**Table 5:** Longitudinal observation of pre-S deletion patterns in HCC patients.

Case	Age(yr)	Genotype	HBeAg	At baseline	2–4 years before HCC	HCC
#7	52	C	−	○—○	<$>\scale130%\raster="rg3"<$>—○	<$>\scale130%\raster="rg3"<$>—○
#26	47	C	<$>\vskip-0.54\scale60%\raster="rg2"<$>	○—<$>\scale130%\raster="rg3"<$>	○—<$>\scale130%\raster="rg3"<$>	○—<$>\scale130%\raster="rg3"<$>
#41	57	C	−	○—○	○—<$>\scale130%\raster="rg3"<$>	○—<$>\scale130%\raster="rg3"<$>
#52	50	C	−	○—○	○—○	<$>\scale130%\raster="rg3"<$>—○
#69	60	B	−	<$>\scale130%\raster="rg3"<$>—○	<$>\scale130%\raster="rg3"<$>—○	<$>\scale130%\raster="rg3"<$>—○
#74	57	C	−	○—<$>\scale130%\raster="rg3"<$>	<$>\scale130%\raster="rg3"<$>—<$>\scale130%\raster="rg3"<$>	<$>\scale130%\raster="rg3"<$>—<$>\scale130%\raster="rg3"<$>
#76	49	C	<$>\vskip-0.54\scale60%\raster="rg2"<$>	○—<$>\scale130%\raster="rg3"<$>	○—<$>\scale130%\raster="rg3"<$>	○—<$>\scale130%\raster="rg3"<$>
#85	39	C	−	○—○	<$>\scale130%\raster="rg3"<$>—<$>\scale130%\raster="rg3"<$>	<$>\scale130%\raster="rg3"<$>—<$>\scale130%\raster="rg3"<$>
#89	47	C	−	○—○	Negative PCR product	○—<$>\scale130%\raster="rg3"<$>
#92	51	C	<$>\vskip-0.54\scale60%\raster="rg2"<$>	○—<$>\scale130%\raster="rg3"<$>	○—<$>\scale130%\raster="rg3"<$>	○—<$>\scale130%\raster="rg3"<$>
#96	45	C	−	○—<$>\scale130%\raster="rg3"<$>	○—<$>\scale130%\raster="rg3"<$>	○—<$>\scale130%\raster="rg3"<$>

○—○, pre-S1—pre-S2; ○, wild type; <$>\scale130%\raster="rg3"<$>, deletion mutation type.

## Discussion

In this case-control study, we compared the virologic differences in pre-S/S regions between 96 HCC patients and 97 age-matched chronic hepatitis subjects. We found a higher frequency of pre-S deletions and pre-S2 start codon mutations in HCC patients. Stepwise multiple logistic regression revealed that pre-S deletions and pre-S2 start codon mutations were the independent risk factors for the development of HCC. In the present study, the prevalence of HBV pre-S deletions in HCC patients and control subjects was 29.2% and 11.3%, respectively, with an overall prevalence of 20.2%. Our findings were in accordance with those reported in other Asian countries [14], [20]. The 5′ terminus of pre-S2 was the favored site for the deletion mutations and the prevalence was significantly higher in HCC than the controls. Previous studies showed that pre-S deletions were more frequent among carriers of HBV genotype C than among carriers of genotype B [14], [15]. However, an analysis of samples from 12 countries, including Vietnam, Nepal, Myanmar, China, Korea, Thailand, Japan, Ghana, Russia, Spain, USA and Bolivia, showed that the prevalence of pre-S deletions in genotype C was similar to that in genotype B [21]. The prevalence of pre-S deletions in this study was not significantly different between genotypes C and B [36/181(19.9%) vs. 3/12(25%), P = 0.669]. This is probably due to the limited number of subjects with genotype B infection in Qidong.

The pre-S1 and pre-S2 regions play an essential role in the interaction with immune responses because they contain several epitopes for T or B cells [22], [23]. Pre-S deletion decreases the expression of surface proteins of HBV, resulting in intracellular accumulation of HBV envelope proteins and viral particles, formation of ground glass hepatocytes, inducing endoplasmic reticulum stress and oxidative DNA damage, and eventually hepatocellular carcinogenesis [12], [15]. In the present study, pre-S2 deletions were detected in a higher frequency than pre-S1 deletions in HBV isolates among HCC patients, which was consistent with the findings from other studies [24], [25]. Therefore, N-terminal half of pre-S2 seemed to be the preferred target region of deletion. Several well-known B- and T-cell epitopes overlap with this region. Among them, the B-cell epitope at amino acids 120 to 145 was most frequently deleted, followed by the T-cell epitope at amino acids 140 to 149. In addition, we also determined which of the well known functional domains of HBV were potentially affected by these deletions. The domains most frequently involved were the transactivator domain in pre-S2 (amino acids 120 to 172) and the polymerized human serum albumin (PHSA)-binding site (amino acids 122 to 135). The pre-S2 deletions have been reported to display tumor-promoting phenotypes in Huh7 cells, for example, enhanced proliferation and clonal expansion abilities, and to cause strong oxidative stress and overall genomic instability, the induced genomic instability surely enhances HCC development, and this may be related with the oncogenic properties of this mutant virus [12], [26], [27]. This suggested the higher oncogenic potential of pre-S2 deletions, compared with that of pre-S1 deletions. Most previous studies on the relationship between pre-S deletion and the risk of HCC were conducted with the use of samples taken either at the baseline of the prospective cohort or after the diagnosis of cancer. Because most HBV mutations are acquired during the course of chronic infection rather than being obtained from an initial infection, it is important to know when or at which stage of the disease the mutations developed [10]. This study was facilitated by the availability of prospectively collected plasma samples from Qidong. To date, there is a lack of longitudinal observation on pre-S deletion over the period of HCC progression. We then recruited a series of serum samples spanning the years before and after HCC diagnosis. Our longitudinal study on patients who carried the pre-S deletion mutations at the stage of HCC also revealed that, in about half of cases, the deletion was indeed absent 5–10 years prior to the occurrence of HCC. The result demonstrated that a gradual occurrence of pre-S deletions during the progression of HCC. This observation provided direct evidence that the pre-S deletions were not acquired at the beginning of infection, but that these mutations arose de novo during the progression of liver disease.

The influence of other types of mutations in pre-S/S genes related to HCC remains uncertain. It has been reported that infection by pre-S2 defective HBV and mutations in the α-determinant of the S gene were often associated with HCC or end-stage liver disease [28], [29]. Pre-S2 start codon mutations may abrogate the expression of M, resulting in pre-S2 defective variants [30]. Pre-S2 defective HBV with point mutations in the pre-S2 ATG have been isolated from patients with fulminant hepatitis [13]. Such mutations have also been reported to be associated with advanced liver disease, including HCC [24]. In our study, pre-S2 start codon mutations were detected in HBV isolates from both HCC and non-HCC patients, pre-S2 start codon mutation was an independent risk factor for the development of HCC. Further more, there was a significant difference in amino acid substitutions at codon 22 in pre-S2 gene between patients with and those without HCC. We also observed that control subjects showed a significant higher frequency of the synonymous substitution of T53C in pre-S2 region than case patients. However, HCC patients and those control subjects did not significantly differ in mutations of the pre-S1 and S regions. These amino acid substitutions in pre-S and S genes related to HCC have rarely been reported. Thus, further studies are needed.

In previous studies, genotype C was reported to be associated with an increased risk of HCC compared with genotype B [6], [31]. However, such a correlation was not observed in this study from Qidong probably due to the limited number of patients with genotype B infection. Additionally, our case-control study revealed that positivity of HBeAg was not an independent risk factor for the development of HCC. Consistent with the REVEAL study, this result indicated that the most likely role of HBeAg as a marker of active viral replication was associated with the increased risk of HCC. Meanwhile, a significant association between elevated serum HBV DNA levels and increased risk of HCC was not observed because it was well established that the level of viremia declines over the course of HBV infection, especially during the period of cirrhosis and HCC.

The strengths of this study include that we used HBsAg carriers not receiving antiviral therapy who were identified through routine physical examination rather than clinical patients, and thus the data are important in understanding the role of viral sequence variation in the natural history of HBV. Furthermore, a longitudinal observation of pre-S deletions during follow-up clearly revealed the evolution of pre-S deletion patterns emerged during the development of HCC. There are also some limitations that should be considered. First, we only used a case-control study with 193 males to validate the associations between HBV pre-S/S mutations and HCC. A prospective cohort study with a large number of HBV pre-S/S mutation-infected patients and a long period of follow-up will better assess the interplay between such mutations and HCC. Second, the direct sequencing method only revealed the predominant strains in the host, it may underestimate the real mutation level in patients, for in most cases, HBV pre-S deletions coexisted with the wild-type HBV. Third, other at-risk mutations (such as V1753, T1762/A1764, T1766, and A1768 mutations in BCP and T1653 mutations in box α of Enhancer II) were not evaluated in this study. Finally, the generalizability of the results is limited because all the study subjects were males, and a larger cohort with longer term follow-up is needed in females. Because there were several limitations existing in the current study, our results should be interpreted with caution and likewise the conclusions of this study should also be drawn cautiously. Therefore, future studies or analyses assessing the risk of HBV pre-S/S mutations on occurrence of HCC should be performed on the basis of overcoming such limitations.

In conclusion, our present study showed that HBV pre-S deletions, especially pre-S2 deletions, were associated with the development of HCC, irrespective of age, HBeAg status, and HBV genotype. Further prospective studies are needed to confirm the role of these mutations in the development of HCC.

## Supporting Information

Table S1 **Original data source for analysis.**
(XLS)
